# Modified Single-Puncture Temporomandibular Joint (TMJ) Arthrocentesis Using a Spinal Needle: A Case Report

**DOI:** 10.7759/cureus.108524

**Published:** 2026-05-08

**Authors:** Asish K Das, Subhasish Burman, Abhijit Maji, Swagatam Samanta, Purbalee Barman, Moumita Ghosh

**Affiliations:** 1 Oral and Maxillofacial Surgery, Dr. R. Ahmed Dental College and Hospital, Kolkata, IND

**Keywords:** arthrocentesis, lavage, single puncture, spinal needle, tmj

## Abstract

Arthrocentesis represents a minimally invasive intervention for managing inflammatory temporomandibular joint (TMJ) disorders. Although the conventional double-needle technique has long been considered the standard approach, recent single-puncture modifications aim to minimize procedural morbidity. This report delineates a refined single-puncture approach utilizing a dual spinal needle assembly. The proposed refinements enable efficacious lytic irrigation, enhance operative efficiency, and confer distinct technical advantages relative to prior single-puncture methods. In this report, a 26-year-old male presented with left-sided preauricular pain and restricted maximal interincisal opening (2 cm). He reported a five-year history of left TMJ clicking, which progressed over three months to a persistent dull ache exacerbated by mandibular excursion and mastication. Examination revealed mandibular deviation to the left on opening and marked preauricular tenderness, suggestive of TMJ pathology.

## Introduction

Temporomandibular joint (TMJ) arthrocentesis, first described by Nitzan et al. in 1991 [1], has become a cornerstone therapy for temporomandibular disorders refractory to conservative measures, including physical therapy, occlusal splints, pharmacotherapy, and behavioral interventions. As a minimally invasive outpatient procedure, it entails irrigation of the superior joint compartment to achieve hydropneumatic lysis of adhesions, thereby alleviating pain and restoring mandibular excursion [1].

The conventional double-needle technique requires precise triangulation for dual capsular punctures: one needle delivers irrigant inflow, the other facilitates outflow. Although effective, this approach demands technical proficiency; blind insertion of the outflow needle often necessitates repeated attempts, heightening risks of extra-articular extravasation and diminishing intra-articular hydraulic pressure essential for adhesion lysis.

This case report presents a modified single-puncture technique employing dual 21-gauge spinal needles, ubiquitous in clinical practice, to establish independent inflow and outflow via a solitary entry point. This configuration minimizes capsular trauma while ensuring efficacious lavage. The method adapts the peripheral intravenous catheter approach of Datarkar et al. (2023) [2] by substituting spinal needles for enhanced adaptability.

## Case presentation

A 26-year-old male presented to the Department of Oral and Maxillofacial Surgery, Dr. R. Ahmed Dental College and Hospital, with a complaint of left preauricular pain and restricted maximal interincisal opening (MIO). He reported a five-year history of left TMJ clicking, progressing to a three-month dull ache exacerbated by wide excursion and mastication.

The patient had previously undergone conservative management in the Department of Prosthodontics; however, symptoms persisted despite a minimum of three months of treatment with occlusal splints, analgesic therapy, and physiotherapy. In view of the refractory nature of the condition, the patient was subsequently referred to the Department of Oral and Maxillofacial Surgery for further management.

**Figure 1:**
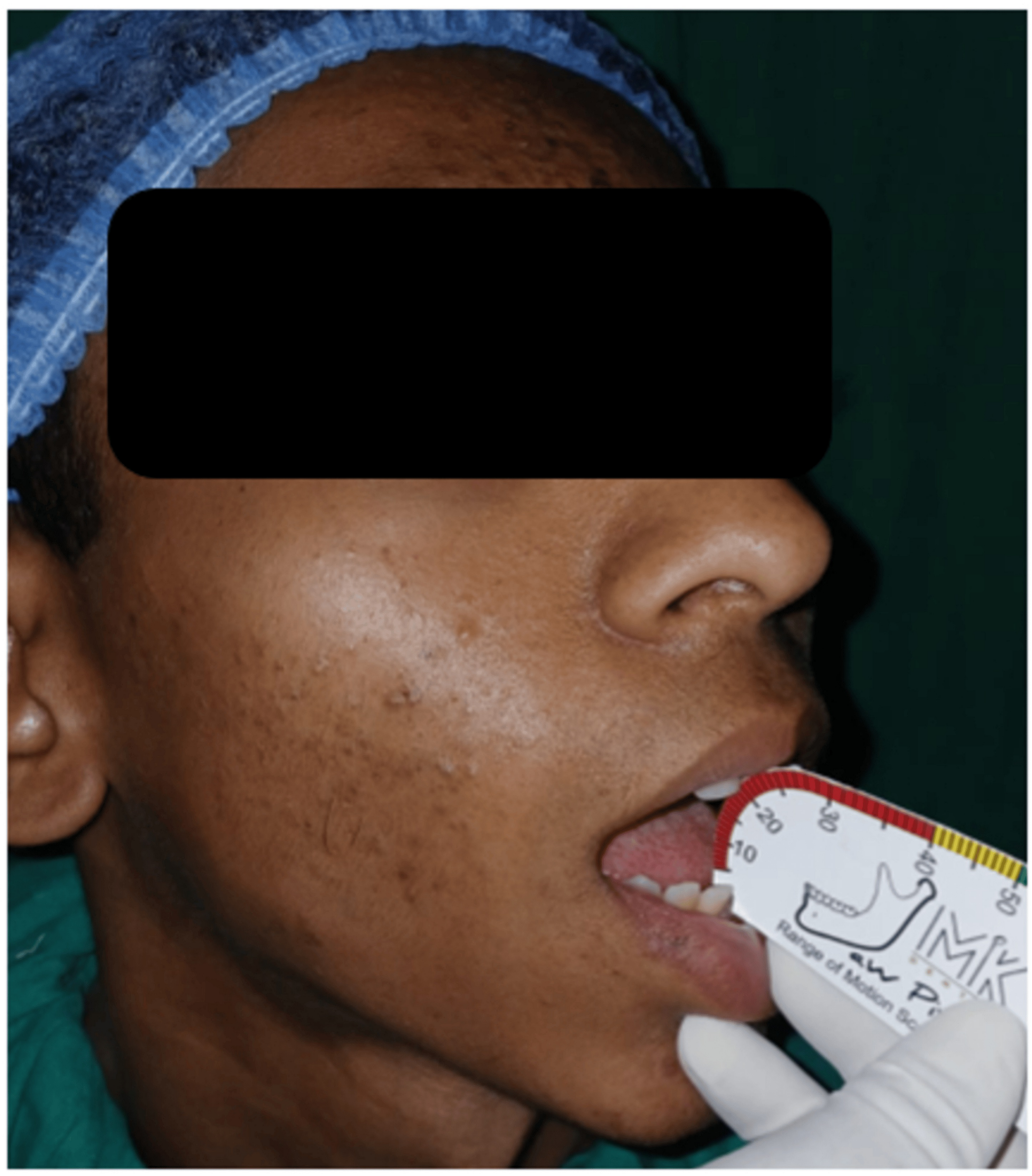
Preoperative maximum incisal opening (MIO) of 2 cm. Written informed consent was obtained from the patient for publication of this case report and accompanying images.

Clinical examination in the Department of Oral and Maxillofacial Surgery revealed an MIO limited to 2 cm (Figure 1), accompanied by leftward mandibular deviation and marked preauricular tenderness. The patient also reported intermittent painful clicking and episodes of joint locking, associated with restricted and painful mandibular function. Cone-beam computed tomography (CBCT) demonstrated normal condylar morphology without evidence of osseous pathology. Magnetic resonance imaging (MRI) could not be obtained due to financial constraints. Based on the aforementioned clinical findings and radiographic assessment, a provisional diagnosis of stage III TMJ internal derangement, according to the Wilkes classification, was established [2].

Initial conservative management consisted of oral administration of aceclofenac (100 mg) combined with thiocolchicoside (4 mg) three times daily for five days, in conjunction with a structured regimen of mandibular mobilization exercises. This approach resulted in a significant reduction in pain; however, residual functional limitation persisted after one week. Given the patient’s prior history of prolonged conservative therapy for approximately three months in the Department of Prosthodontics without meaningful clinical improvement, a decision was made to proceed with TMJ arthrocentesis under local anaesthesia for a better outcome.

The procedural armamentarium comprised two 21-gauge spinal needles, lactated Ringer’s solution as the irrigating medium, and 20 mL syringes for joint lavage. Local anesthesia was administered using 2% lidocaine, and aseptic preparation was achieved with 10% povidone-iodine solution. An intra-articular injection of triamcinolone acetonide (40 mg) was subsequently delivered at the end of the lavage. Anatomical landmarks were delineated using surgical skin markers in conjunction with a calibrated ruler, while the mandibular range of motion was assessed using a jaw range-of-motion scale. An endodontic silicone stopper was employed as an adjunct for procedural precision, and sterile gauze was utilized to maintain a controlled operative field.

Two 21-gauge spinal needles were employed in this technique. Reference markings were placed at 25 mm and 45 mm on each needle using a sterile surgical marker. The 25-mm mark denoted the maximum allowable depth for intra-articular penetration to ensure procedural safety and prevent over-insertion.

**Figure 2:**
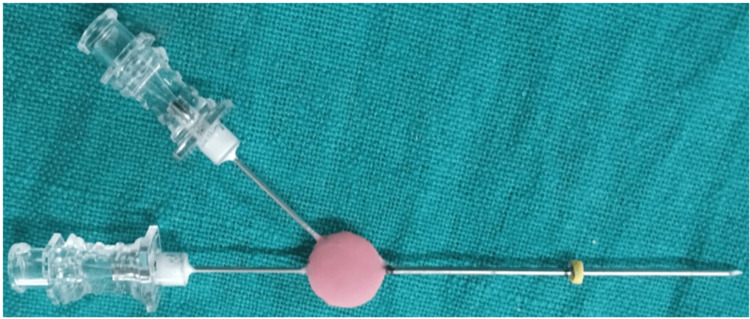
Two 21-gauge spinal needles were rigidly coupled distal to the 45-mm reference mark using self-polymerizing acrylic resin, creating a single unified instrument. At the level of the 45-mm marking, one needle was deliberately contoured to a 45° angulation to facilitate improved intraoperative maneuverability, while the second needle was preserved in a straight configuration to enhance handling stability and to optimize bidirectional inflow-outflow dynamics during lavage. An endodontic silicone stopper was positioned at the 25-mm mark on the needle assembly to ensure accurate depth control and to mitigate the risk of inadvertent over-penetration.

The needles were rigidly coupled beyond the 45-mm reference point using self-polymerizing acrylic resin, thereby forming a single, integrated instrument. At the 45-mm marking, one needle was contoured to a 45° angulation to facilitate ease of manipulation [2], whereas the second needle was maintained in a straight configuration to enhance handling characteristics and optimize bidirectional inflow-outflow dynamics during lavage (Figure 2).

**Figure 3:**
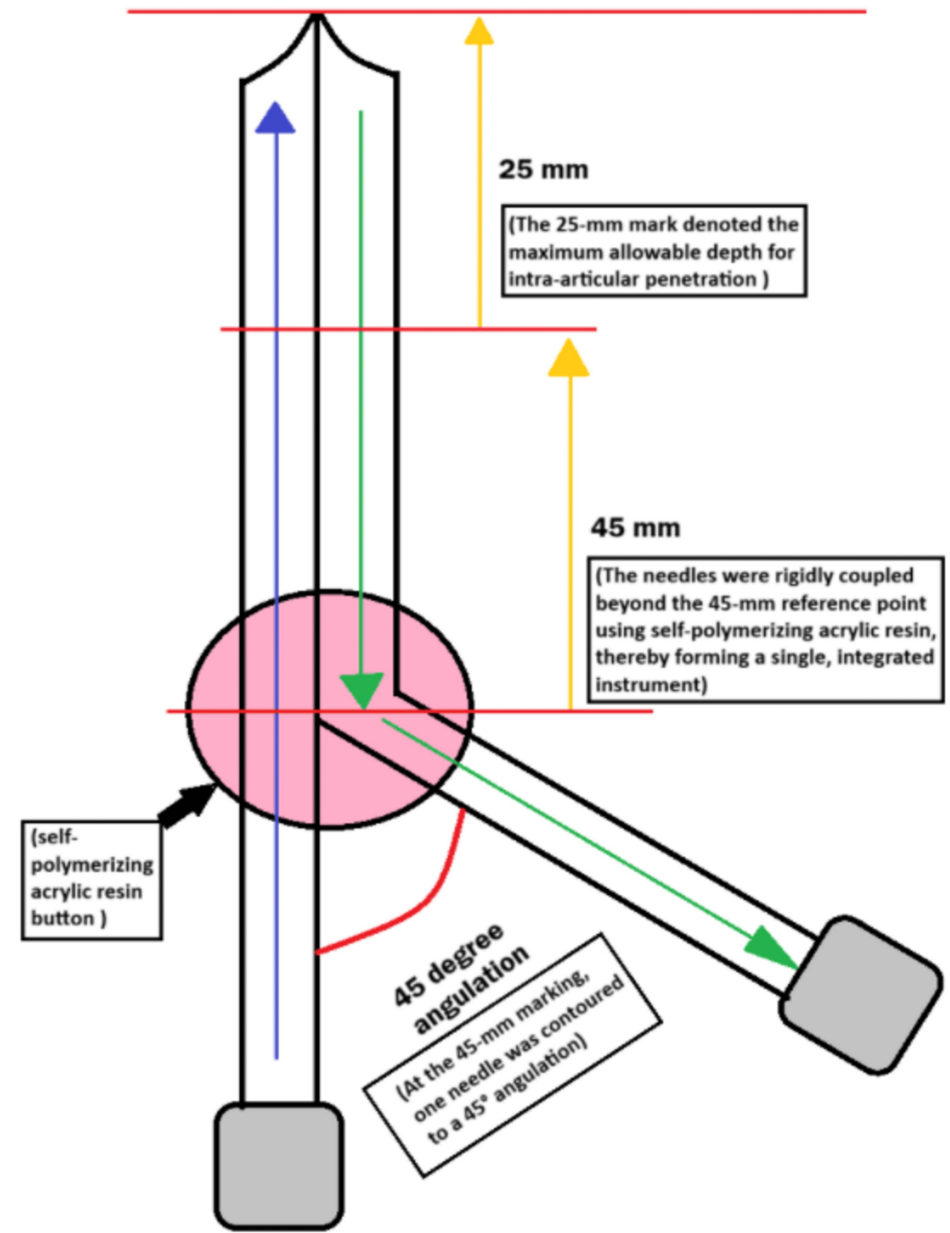
Schematic representation of the instrument. Two 21-gauge spinal needles were pre-marked at 25 mm and 45 mm to regulate insertion depth, with 25 mm serving as the maximum safe intra-articular limit. The needles were rigidly coupled beyond the 45-mm mark using self-polymerizing acrylic resin to form a single unit; one needle was angulated to 45° for improved maneuverability, while the other remained straight to facilitate controlled bidirectional lavage. Blue and green arrows indicate the bidirectional flow dynamics. Image credit: The schematic diagram was created by the author using the Microsoft Paint application (Microsoft Corp., Redmond, WA, USA).

A schematic representation of the modified needle assembly is provided to facilitate clear visualization and enhance comprehension of its structural configuration and functional design (Figure 3).

Prior to clinical use, the assembled device was sterilized with ethylene oxide gas in accordance with standard infection-control protocols.

Procedure

The preauricular region overlying the TMJ was aseptically prepared with a topical antiseptic solution and appropriately draped to maintain a sterile operative field.

**Figure 4:**
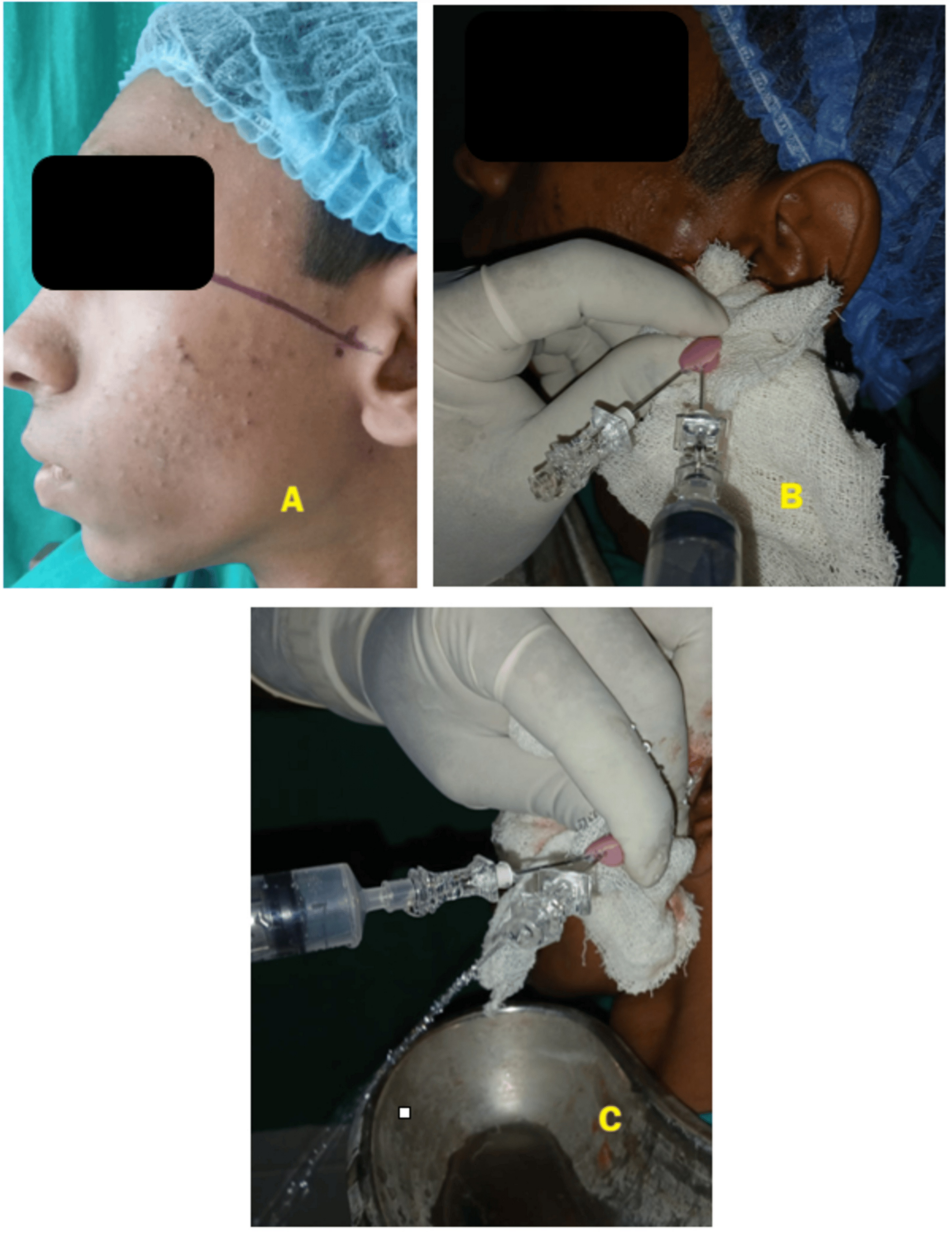
Intraoperative images of temporomandibular joint (TMJ) arthrocentesis. A: Surface anatomical landmarks were delineated by drawing a line from the lateral canthus to the most posterior and central point of the tragus (Holmlund-Hellsing line). B: The needle was introduced into the superior joint compartment at the predetermined entry point. C: Lavage was performed. Written informed consent was obtained from the patient for publication of this case report and accompanying images.

Following administration of an auriculotemporal nerve block, the joint space was insufflated with 2 mL of a lidocaine-normal saline solution to facilitate capsular distension and confirm intra-articular access. Surface anatomical landmarks were delineated by drawing a line from the lateral canthus to the most posterior and central point of the tragus (Holmlund-Hellsing line; Figure 4A). The needle entry point was established along this canthotragal line, approximately 10 mm anterior to the midpoint of the tragus and 2 mm inferior to the reference line, corresponding to the superior joint compartment and the needle is introduced into the superior joint compartment at the predetermined entry point (Figure 4B), with its trajectory oriented in a superior, anterior, and medial direction to facilitate precise intra-articular access [3]. The mean distance from the cutaneous surface to the center of the superior joint space is approximately 25 mm. An endodontic silicone stopper was secured on the needle to provide precise depth control and prevent over-penetration.

Subsequently, approximately 300 mL of Ringer’s lactate solution was infused into the superior joint space using a 10-mL syringe. Continuous lavage was accompanied by gentle manual manipulation of the mandible (Figure 4C). To achieve adequate intra-articular hydraulic distension prior to establishing effective outflow, the needle bevels were oriented such that the tips remained closely approximated, while the bevel apertures faced in opposite directions to optimize inflow-outflow dynamics.

Upon completion of lavage, the needles were withdrawn, and the mandible was gently mobilized in vertical, protrusive, and lateral excursions to promote disc release and disrupt intra-articular adhesions. The patient was subsequently managed according to the same standardised postoperative protocol.

**Figure 5:**
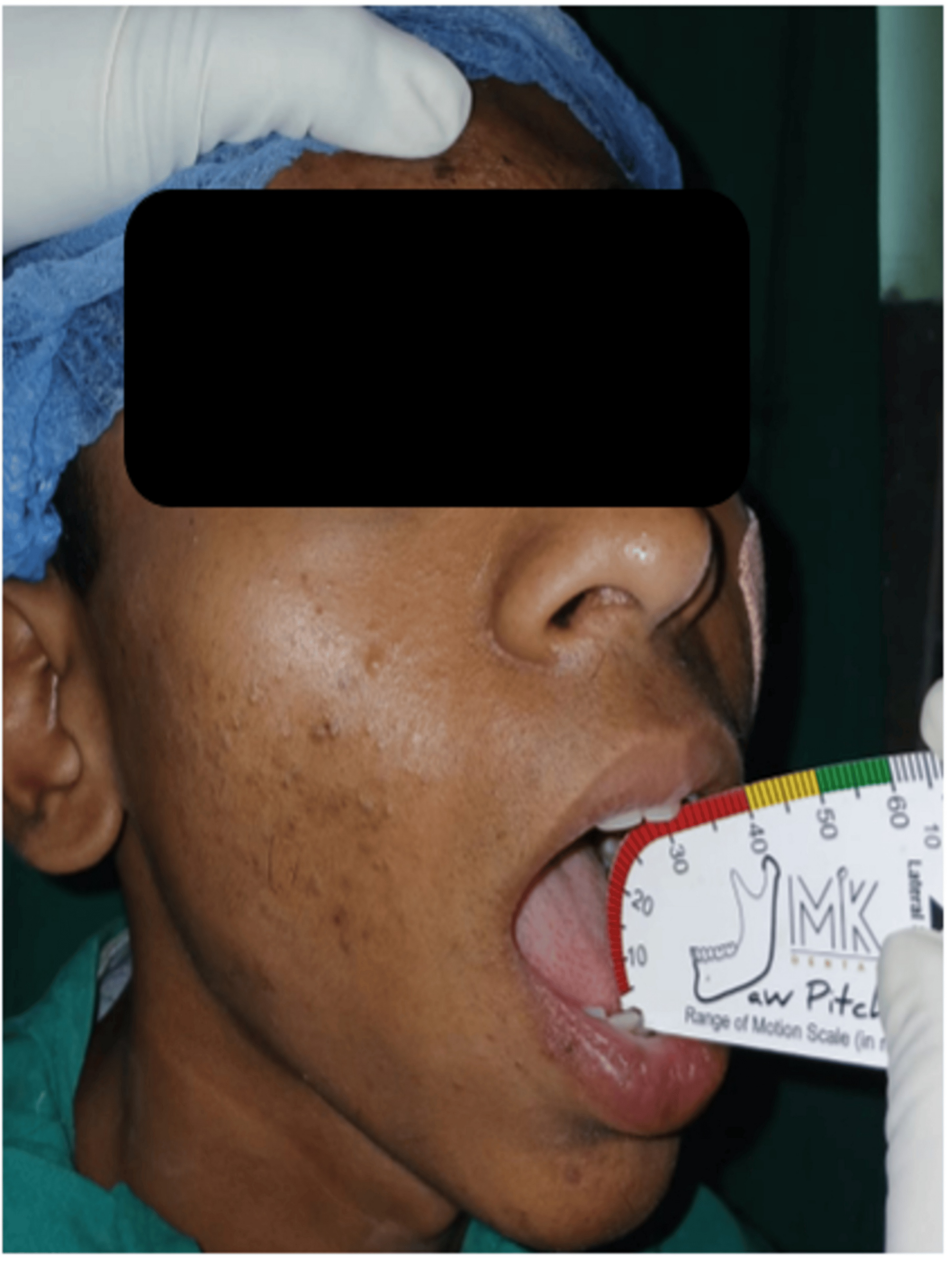
Maximum incisal opening (MIO) of 3 cm at the first postoperative week. Written informed consent was obtained from the patient for publication of this case report and accompanying images.

Pre-operative and post-operative outcomes were assessed using pain intensity scores in the Visual Analogue Scale (VAS) and MIO measurements at the first week post-operatively, second week post-operatively, fourth week post-operatively, and eighth week post-operatively. Clinical evaluation revealed a substantial reduction in pain levels, accompanied by progressive improvement in mandibular function. Notably, significant gains were observed as early as the first postoperative week. The pre-operative MIO of 2.0 cm increased to 3.0 cm at the first post-operative week (Figure 5), 3.5 cm at the second post-operative week, 4.0 cm at the fourth post-operative week, and 4.2 cm at the eighth post-operative week follow-up examination (Table 1).

**Table 1:** Maximum incisal opening (MIO) and pain assessed using the Visual Analogue Scale (VAS) during the preoperative and postoperative periods.

Parameters	Pre-operative period	First-week post-operative period	Second-week post-operative period	Fourth-week post-operative period	Eighth-week post-operative period
MIO (in cm)	2	3	3.5	4	4.2
Pain in the VAS scale	7	2	0	0	0

## Discussion

The conceptual foundation of TMJ arthrocentesis and lavage derives from the clinical success of TMJ arthroscopy, which demonstrated not only diagnostic value but also significant therapeutic efficacy. Arthroscopic intervention was shown to yield substantial improvements in pain intensity, mandibular range of motion, and overall joint function in appropriately selected patients. These benefits were largely attributed to lavage of the superior joint compartment, with consequent removal of inflammatory mediators and mechanical disruption of intra-articular adhesions.

Contemporary understanding of TMJ arthrocentesis recognizes multiple interrelated therapeutic mechanisms. These include irrigation of the superior joint space, generation of intra-articular hydraulic pressure, and controlled mandibular manipulation aimed at releasing adhesions and overcoming the so-called “anchored disc phenomenon” or suction-cup effect. Collectively, these mechanisms facilitate restoration of disc mobility and functional mandibular movement. In selected cases, adjunctive intra-articular corticosteroid administration may further potentiate anti-inflammatory effects and enhance clinical outcomes [4-6].

The findings observed in the present case report are consistent with early clinical investigations demonstrating that TMJ arthrocentesis combined with lavage and mandibular manipulation constitutes an effective therapeutic modality for acute persistent closed lock. Reported outcomes include significant increases in maximal mouth opening, improvement in mandibular function, and substantial reduction in pain scores. Beyond its established role in the management of acute closed lock, TMJ arthrocentesis and lavage have also been advocated for other intra-articular pathologies, including osteoarthritis, early-stage rheumatoid arthritis, and acute intracapsular trauma associated with TMJ hemarthrosis [7-9].

Although generally considered a minimally invasive and safe procedure, TMJ arthrocentesis is not devoid of potential complications. Reported adverse events include extravasation of irrigating solution into adjacent soft tissues; transient or, rarely, permanent injury to the facial nerve (incidence approximately 0.6-0.7%); sensory disturbances involving branches of the trigeminal nerve (0.1-2.4%); and otologic complications (0.5-8.6%). Additional local and regional sequelae described in the literature encompass preauricular hematoma, superficial temporal artery aneurysm, arteriovenous fistula formation, transarticular perforation, intracranial perforation with resultant extradural hematoma, parapharyngeal space edema, and other intra-articular complications [10-13].

Şentürk and Cambazoğlu proposed a classification system for TMJ arthrocentesis techniques based on the number of puncture sites employed, distinguishing between single-puncture arthrocentesis (SPA) and double-puncture arthrocentesis [12]. Within the SPA category, techniques are further subclassified according to the number of needles or lumens utilized: type 1, involving a single-needle cannula technique, and type 2, employing either a dual-needle configuration or a double-lumen cannula introduced through a single puncture site [14]. The technique described in the present case corresponds to the type 2 SPA approach; however, it uniquely incorporates two spinal needles rather than a conventional double-lumen cannula.

The modified spinal needle-based technique presented herein represents a simple, cost-effective, and reproducible method for single-puncture TMJ arthrocentesis. A principal advantage lies in the utilization of standard spinal needles, which are routinely used for lumbar puncture and are therefore widely available in most healthcare settings worldwide, rendering the approach economically advantageous and readily adoptable.

Furthermore, the single-puncture design minimizes capsular violation and soft tissue trauma by limiting joint access to a solitary entry point, thereby reducing procedural complexity and potential morbidity. The incorporation of separate inflow and outflow channels facilitates efficient and continuous lavage of the superior joint compartment. In addition, the 45° angulation of one needle enhances intra-articular access, improves device stability, and allows for more controlled manipulation within the joint space, thereby optimizing procedural efficacy.

## Conclusions

TMJ lysis and lavage principally target synovitis resolution, adhesion rupture, analgesia, and mobility restitution. Arthrocentesis offers a safe, minimally invasive recourse for internal derangement manifesting as a closed lock. This report underscores the merits of our modified single-puncture spinal needle technique: procedural simplicity, attenuated capsular trauma, efficacious bidirectional lavage, and enhanced intra-articular access/stability via angulated configuration, positioning it as a pragmatic advancement in TMJ intervention. Further research is warranted to comprehensively elucidate the relative advantages and limitations of these techniques, to determine the true clinical efficacy of modified SPA, and to facilitate direct comparison with the conventional approach. Future studies should incorporate larger, standardized sample populations, utilize similar instrumentation, and include extended follow-up periods to ensure robust and generalizable outcomes.

## References

[1] Nitzan DW, Dolwick MF, Martinez GA (1991). Temporomandibular joint arthrocentesis: a simplified treatment for severe, limited mouth opening. J Oral Maxillofac Surg.

[2] Datarkar A, Purohit S, Relan P (2023). Novel modification of the use of peripheral intravenous catheters for single puncture arthrocentesis of temporomandibular joint: a technical note. J Maxillofac Oral Surg.

[3] Guarda-Nardini L, Manfredini D, Ferronato G (2008). Arthrocentesis of the temporomandibular joint: a proposal for a single-needle technique. Oral Surg Oral Med Oral Pathol Oral Radiol Endod.

[4] Talaat W, Ghoneim MM, Elsholkamy M (2016). Single-needle arthrocentesis (Shepard cannula) vs. double-needle arthrocentesis for treating disc displacement without reduction. Cranio.

[5] Sanders B (1986). Arthroscopic surgery of the temporomandibular joint: treatment of internal derangement with persistent closed lock. Oral Surg Oral Med Oral Pathol.

[6] Rehman KU, Hall T (2009). Single needle arthrocentesis. Br J Oral Maxillofac Surg.

[7] Alkan A, Baş B (2007). The use of double-needle canula method for temporomandibular joint arthrocentesis: clinical report. Eur J Dent.

[8] Emshoff R, Rudisch A (2003). Are internal derangements and osteoarthrosis linked to changes inclinical outcome measures of arthrocentesis of the temporomandibular joint?. J Oral Maxillofac Surg.

[9] Nishimura M, Segami N, Kaneyama K, Sato J, Fujimura K (2004). Comparison of cytokine level in synovial fluid between successful and unsuccessful cases in arthrocentesis of the temporomandibular joint. J Oral Maxillofac Surg.

[10] Carlsson GE (1999). Epidemiology and treatment need for temporomandibular disorders. J Orofac Pain.

[11] Greene CS (2001). The etiology of temporomandibular disorders implications for treatment. J Orofac Pain.

[12] Şentürk MF, Cambazoğlu M (2015). A new classification for temporomandibular joint arthrocentesis techniques. Int J Oral Maxillofac Surg.

[13] Tozoglu S, Al-Belasy FA, Dolwick MF (2011). A review of techniques of lysis and lavage of the TMJ. Br J Oral Maxillofac Surg.

[14] Yongvikul A, Kim JY, Ku JK, Jung JH, Huh JK (2024). Needle orientation for temporomandibular joint arthrocentesis in Koreans. Cranio.

